# Machine learning approach for prediction of TBM performance and risk of jamming in Himalayan geology using a cross-project tunnelling database

**DOI:** 10.1038/s41598-025-34273-z

**Published:** 2026-01-08

**Authors:** Tek Bahadur Katuwal, Krishna Kanta Panthi, Chhatra Bahadur Basnet

**Affiliations:** 1 Norwegian University of Science and Technology (NTNU), Trondheim, Norway; 2 Tribhuvan University, IOE, Pashchimanchal Campus, Pokhara, Nepal

**Keywords:** Himalayan geology, TBM performance, Machine learning, SHAP, Jamming risk, Engineering, Solid Earth sciences

## Abstract

Tunnel Boring Machine (TBM) excavation in the Himalayan region presents significant challenges due to complex geological conditions. The identification of tunnelling risk under such conditions is crucial for optimizing TBM performance. This study proposes a machine learning (ML)-based framework to predict TBM performance and assess associated jamming risk using a cross-project TBM database from the Himalayan region. The study employs ML approaches, including random forest, bagging, XGBoost, stacking ensemble, and artificial neural network. The combined stratified cross-project database results in improved model performance, with R² values ranging from 0.960 to 0.965. Shapley Additive exPlanations (SHAP) analysis revealed that the TBM net penetration rate (PRnet) is primarily influenced by response parameters such as torque and thrust with corresponding rock mass quality conditions. A combined jamming risk (CJR) scoring system was found to be useful to predict potential TBM jamming events in tunnelling. The CJR system effectively provides early warning signals in at least one ring (~ 1.5 m) in advance of TBM stuck events. The findings demonstrate that the application of ML-based techniques offers a valuable tool for predicting TBM performance and jamming risk, enabling adjustment of tunnelling parameters in the Himalayan region and similar complex geological settings worldwide.

## Introduction

The Himalaya was formed by the northward movement of Indian tectonic plate colliding with Eurasian plate, a process that began about 70 − 50 million years ago and continues today^1,2^. Due to the fact of such considerable tectonic activities, the geological and tectonic setting in the Himalayan region is complex, which significantly influences the rock mass quality conditions with higher degree of fracturing, folding, faulting, jointing, weathering, and frequent occurrence of shear/weakness zones^2^. In recent years, Tunnel Boring Machines (TBMs) excavation techniques have been extensively used in tunnelling projects worldwide with advantages of good construction efficiency, safety, better construction quality, and high excavation rate over drill and blast method^3–6^. Double-shield TBMs are currently the preferred choice for tunnel boring in the Himalayan region of Nepal due to their efficiency in handling complex geological conditions^4,7,8^. This is especially relevant for tunnels with limited options for shorter construction adits, particularly for those longer than 10 kilometers^8^. However, geological conditions prevailing in the Himalayan region are highly sensitive and challenging for TBM tunnelling. This is because the rock mass in the Himalayan region exhibits a higher possibility of water ingress, tunnel face collapse, and squeezing, which ultimately leads to the risk of the TBM stuck or jamming^4,9^. These challenges demand careful planning and the use of appropriate tunnelling techniques to mitigate geological risk and maintain optimal tunnelling performance.

The TBM performance forecast during planning and design phases, and optimization during TBM advancement is crucial for precise estimation of time and costs^7,10–12^. Over the past few decades, many researchers have developed various models for predicting TBM performance. These include empirical, theoretical, and statistical models. The precision of empirical models is limited; however, these models are still acceptable and are primarily used for planning and design of TBM tunnelling^10,13^. Statistical models have improved, addressing the limitations of empirical models and increasing their applicability to a wider range of geological conditions to forecast TBM performance. However, these methods lack real-time TBM performance forecasts for longer TBM drives. This is mainly because there is a difficulty in mapping and handling the data recorded by TBM.

Currently, machine learning (ML) techniques have emerged in underground construction due to their high computational efficiency, leading to better prediction accuracy and the flexibility to capture non-linear and complex relationships^7,14–16^. Recent studies have employed ML techniques for various TBM-related tasks, including rock mass classification in double-shield TBMs^7,15^ and open TBMs^14^, as well as lithology classification^17^ and cutter wear prediction^18^ in EPB-TBM excavation, highlighting the expanding role of data-driven approaches in tunnelling. Many researchers have proposed ML models to predict net penetration rate of TBM on the basis of field parameters including intact rock properties and rock mass properties^19–21^. These models show enhanced prediction performance. However, these techniques have not matured due to use of limited number of field datasets. The TBM interacts with the surrounding geology, leading to fluctuations in key operational parameters that reflect ground conditions and rock properties^5,7,14,22^. Since it records large amounts of data each cycle, ML can effectively capture complex and non-linear relationships between TBM performance and geology^12,23^. Motivated by these findings, Zhu et al. (2021)^24^ and Flor et al. (2023)^12^ proposed ML models to predict TBM penetration rate using machine parameters. These models demonstrated good predictive performance; however, they relied solely on project-specific machine parameters while excluding geological factors. Moreover, no single model consistently performs best across all datasets, and applying individual model to unseen data may not exhibit good results^14,25^. Consequently, their applicability to new projects with different characteristics, such as TBM cutterhead diameter, rated torque, thrust, disc cutter number, and geological conditions, may be limited. This highlights the need to evaluate suitable ML algorithms under varying conditions. Keeping this in mind, this manuscript presents well-structured frameworks to predict the TBM performance in Himalayan geology using cross-project tuunelling database. This framework incorporates important aspects such as (a) inclusion of cross-project geological and machine parameters, (b) the integration of geological anomalies and associated TBM parameters, (c) evaluation and comparison of predictive performance of different ML algorithms, (d) model interpretability using the Shapley Additive Explanations (SHAP) tool, and (e) practical implementation of output results to assess TBM jamming risk. To the best of the authors’ knowledge, no study has yet employed a purely ML-based data-driven approach to predict TBM performance while incorporating these specific aspects. While doing so, the following research activities have been performed;

- an improved TBM performance prediction model is developed using the database from cross-project of Nepal Himalaya.
- the model interpretability analysis using the SHAP tool is conducted to assess the impact of the selected features.
- TBM jamming risk assessment is conducted through statistical analysis.
- the empirical guideline for TBM tunnelling control and response parameters ranges corresponding to geological conditions is established using statistical analysis.

Hence, this research has developed a robust TBM performance prediction framework that supports real-time monitoring of jamming risk in the Himalayan region of Nepal and in similar geological conditions around the world.

## Materials and methodology

**Fig. 1:**
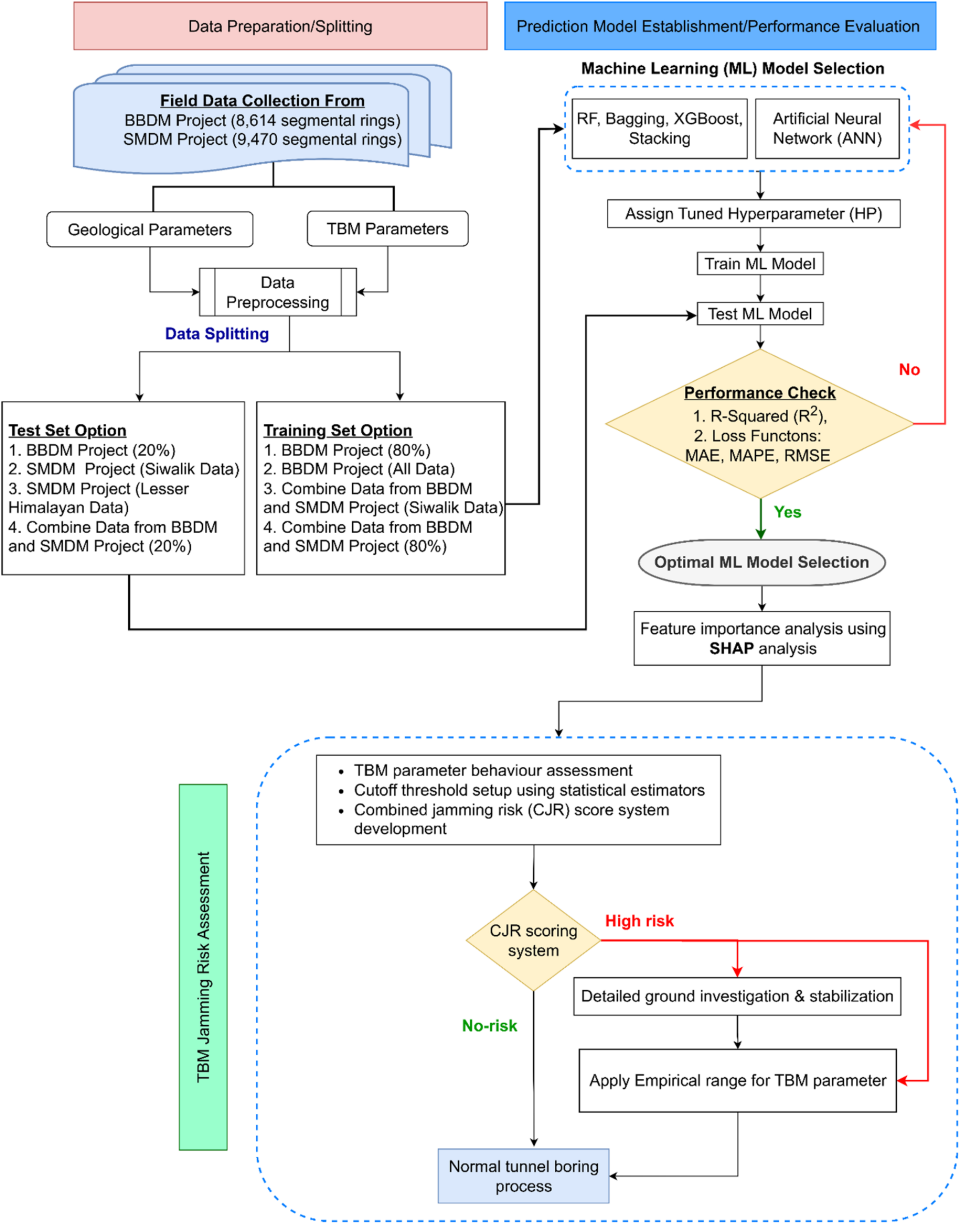
Flowchart of cross-project database utilization for evaluating TBM net penetration rate using ML techniques.

This research aims to develop a well-structured TBM performance prediction framework for the Himalayan region of Nepal using a cross-project database. The framework supports real-time monitoring of jamming risk and assists TBM operators in adjusting machine parameters during tunnel excavation. Figure 1 presents the detailed research workflow. The methodology is organized into two phases. Phase 1 focuses on development of a well structured framework for prediction of TBM net penetration rate using various ML models. This phase comprises six main steps: (1) description of case projects and field data collection, (2) feature selection, (3) data distribution and standardization, (4) model selection and hyperparameter tuning, (5) cross-project model development and evaluation, and (6) model transparency using SHAP analysis, based on the best-performing model. The SHAP analysis identifies the influence of selected input features on the target variable. Phase 2 utilizes the most influential features identified in Phase 1 to assess TBM jamming risk. Statistical analyses were performed to evaluate the potential risk of TBM jamming events under varying geological and operational conditions. Based on these findings, an empirical guideline is developed to support the adaptive adjustment of tunnelling parameters suitable for the TBM tunnelling projects in the Himalayan geological conditions of Nepal. Further, detailed descriptions of each step are presented in the following sections.

### Description of case projects and field data

**Fig. 2:**
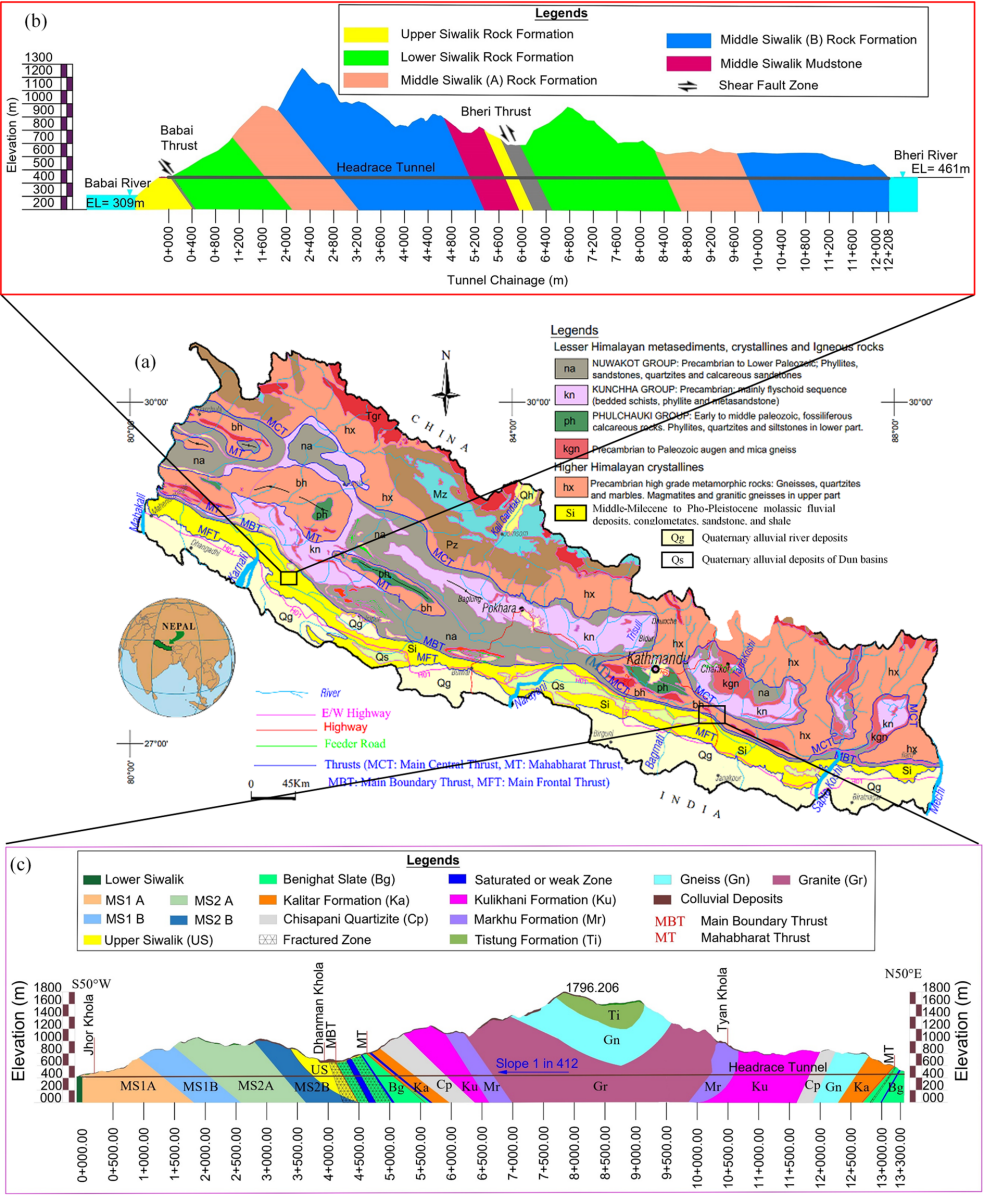
(**a**) Location of projects (Revised from Panthi and Basnet (2019)^28^; GESPA, (2021)^29^. Longitudinal geological profile of tunnel in: (**b**) BBDM project (Revised from Panthi, (2019)^4^; GESPA, (2021)^29^, (**c**) SMDM project (Revised from autocad drawing provided by Government of Nepal, (2025)^30^.

In this research, two inter-basin projects, namely Bheri Babai Diversion Multipurpose (BBDM) project and Sunkoshi Marin Diversion Multipurpose (SMDM) project, are selected as case studies from Nepal. For the first time in Nepal, the 12 km-long headrace tunnel of the BBDM project was excavated using a double-shield TBM with a finished diameter of 4.20 m. The BBDM project is the first inter-basin project located in Surkhet District of Karnali Province in Nepal. A detailed introduction to the project can be found in Katuwal et al. (2024)^7^ and Katuwal and Panthi (2025)^26^. The SMDM project is also inter-basin project located in the Sindhuli district of Bagmati Province in Nepal. The project has a 13.3 km long headrace tunnel with an excavation diameter of 6.40 m (internal diameter of 5.50 m). The excavation of the headrace tunnel began in October 2022 and was completed in May 2024. The excavation breakthrough was achieved 11 months ahead of schedule, which is a record of TBM tunnelling within Himalayan region. The locations of these two projects and longitudinal geological profiles are presented in Fig. 2.

The BBDM project is located in the youngest Siwalik rock formation and is bounded by two major thrusts, namely the Main Frontal Thrust (MFT) and the Main Boundary Thrust (MBT). The headrace tunnel of BBDM project passes through Siwalik rock formation consisting of intercalations of medium to fine-grained sandstone, siltstone, mudstone, and conglomerate rocks (Fig. 2b). On the other hand, the headrace tunnel of SMDM project passes through both Siwalik rock formation and Lesser Himalayan rock formation (Fig. 2c).

As seen in Fig. 2c, the Siwalik rock formation at SMDM project mainly consists of Lower Siwalik (LS), Middle Siwalik1 ‘A’ (MS1 A), Middle Siwalik2 ‘A’ (MS2 A), Middle Siwalik 1 ‘B’ (MS1 B), Middle Siwalik 2 ‘B’ (MS2 B), and Upper Siwalik (US). The headrace tunnel encountered lithological conditions mainly characterized by an intercalation of medium to fine-grained sandstone, siltstone, mudstone, and conglomerate. On the other hand, the Lesser Himalayan part of headrace tunnel encountered a window of Higher Himalaya rock formation consisting Kathmandu Complex and Bhimphedi Group. These equivalent rock formations consist of Benighat Slates (Bg), Kalitar formation (Ka), Chisapani Quartzite (Cp), Kulikhani formation (Ku), Markhu formation (Mr), Granite (Gr), Gneiss (Gn), and Tistung formation (Ti). The Benighat Slates formation consists of schist, quartzite, dolomite, and slate rocks. Chisapani Quartize, Kalitar formation, and Kulikhani formation consist of schists, quartzite, and gneiss rocks. Moreover, Markhu formation consists of schist, quartzite, and calcareous schist rocks. Tistung formation is composed of intercalation of thickly foliated micaceous quartzite with thinly foliated garnet-biotite schist. The Lesser Himalayan part of headrace tunnel passes through amphibolite, gneiss, granite, quartzite, slate, schist, dolomite, and other calcareous rocks^27^. As seen in Fig. 2c, the headrace tunnel also encountered several major and minor faults, folds, shear zones, and a major syncline. The headrace tunnel crosses the MBT, which is approximately 400 m wide and separates the Siwalik rock formation from the Lesser Himalayan rock formation, at a chainage of 4 + 256 m (measured from the Marin/powerhouse side). Moreover, the headrace tunnel crosses the Mahabharat Thrust with a width of 50 m at two locations at a chainage of 5 + 000 m and 13 + 092 m. Likewise, the headrace tunnel traverses through Mahabharat Synclinorium, which consists of granite rock where overburden reaches its maximum of 1,350 m.

The BBDM project was excavated using a double-shield TBM designed with features including probe drilling, stepped shield, difficult ground solution (DGS), and forepoling^7^. The TBM was dismantled following the successful tunnel breakthrough. The same TBM was later re-engineered and employed in the SMDM project. The geological conditions along the headrace tunnel of SMDM project are more challenging due to high overburden, variable rock types, and the presence of major thrust faults, fracture zones, and a major syncline. To address these challenges, the key components of the TBM such as the main body, cutterhead, auxiliary cylinders, and ventilation system were modified. The modified TBM consists of several features such as a tapered shield, an enclosed cutterhead, a high-thrust system, and overcut capabilities (Table 1). The tapered shield was added to reduce the risk of the TBM becoming stuck in squeezing ground conditions, while the enclosed cutterhead minimized the possibility of surrounding rock mass collapse. Similarly, the high-thrust system provided extra thrusting force, enabling the TBM to advance through squeezing ground conditions of the fault zones.

**Table 1:** Specification of TBM used in the Nepal Himalaya.

TBM features	BBDM project	SMDM project
TBM Type	Double shield	Double shield
Excavation diameter (m)	5.06	6.40
Total length of machine (m)	274	274
Cutterhead speed (rpm)	0–11.4.4	0–11.4.4
Rated torque (kNm)	3475	4054
Break out torque (kNm)	5733	6081
Maximum thrust (kN)	20,826	31,220
Number of 17-inch disc cutters	33	41
Power of main drive (kW)	6*330 = 1980	7*330 = 2310
Total power (kW)	3027	3513

Real-time field records of stable-phase TBM control and response parameters with corresponding geological parameters of 8,614 segmental rings from the BBDM project and 9,470 segmental rings from the SMDM project were collected. The resulting TBM database contains mean parameter values for each segment without missing data. The database was then transformed for further use.

### Feature selection

**Fig. 3:**
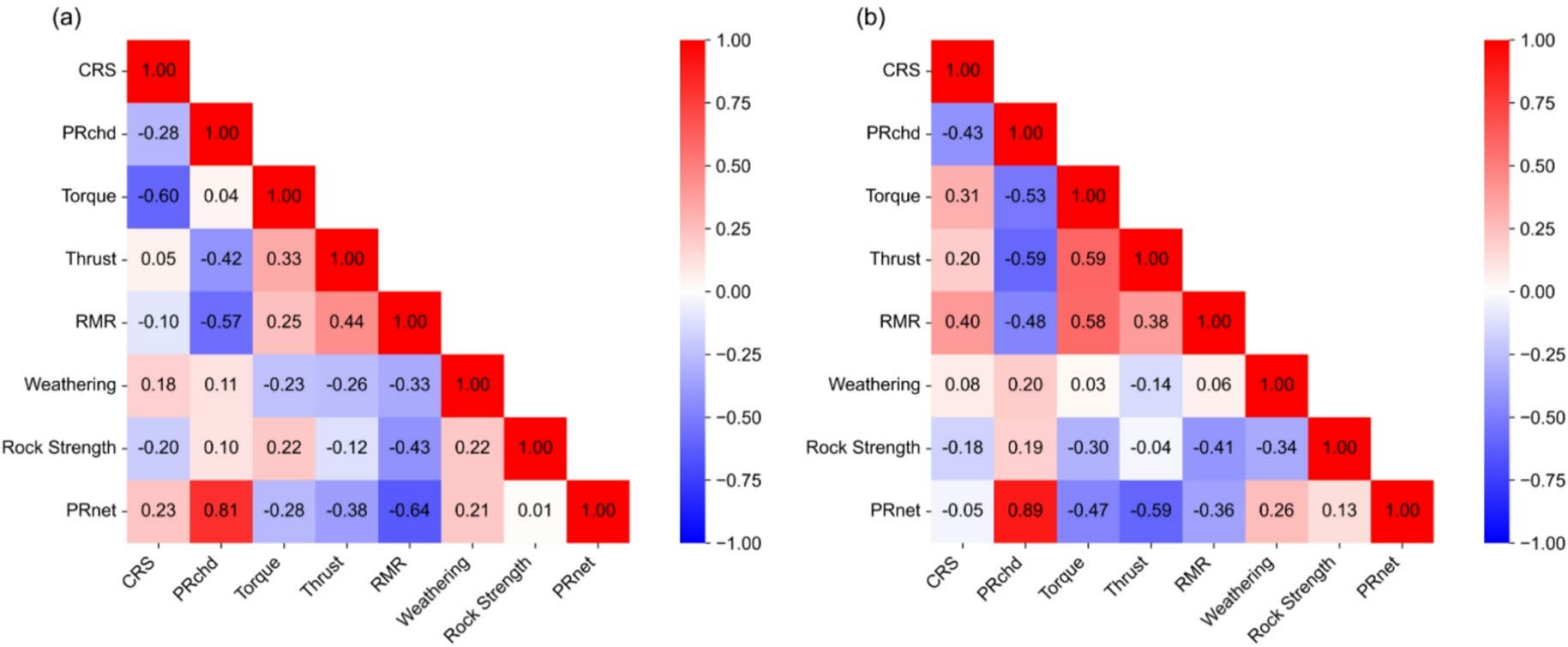
Pearson correlation coefficients between TBM parameters and geological conditions: (**a**) BBDM project, (**b**) SMDM project.

On the basis of TBM boring cycle, many researchers have proposed data-driven approaches that consider key rock-breaking parameters such as cutterhead speed (CRS), cutterhead penetration rate (PRchd), net penetration rate (PRnet), torque, and thrust force, and have identified a good correlation with the corresponding geological conditions^5–7,31,32^. Therefore, these TBM parameters are selected as key rock-breaking parameters. Additionally, TBM parameters fluctuate with corresponding geological conditions such as rock mass rating (RMR), weathering grade, and rock strength and show notable correlations with TBM performance. A detailed discussion regarding the selection of geological parameters and their influence on TBM performance can be found in Katuwal and Panthi (2025)^33^. Thus, these geological parameters are also considered as input features for TBM performance prediction models. The Pearson correlation coefficient (PCC) analysis was used to evaluate the linear correlation between the selected input feature and TBM PRnet. The PCC ranges from − 1 to + 1. A value of + 1 indicates a perfect positive correlation, whereas − 1 indicates a perfect negative correlation. A value of 0 indicates no correlation between the variables. This method is effectively used to enhance the reliability of input features by eliminating redundant parameters. When the correlation coefficient between two parameters is greater than or equal to ± 0.9, one parameter is removed while the other parameter is retained^25,34^. This ensures that multicollinearity between variables does not adversely affect model performance. The correlation between these parameters is presented in Fig. 3.

As illustrated in Fig. 3a, the target variable PRnet in the headrace tunnel of BBDM project exhibits a negative correlation with the input features such as torque, thrust, and RMR, with correlation coefficients of −0.28, −0.38, and − 0.64, respectively. Conversely, PRnet shows a positive correlation with CRS, PRchd, weathering grade, and rock strength, with corresponding values of 0.23, 0.81, 0.21, and 0.01. In the case of the headrace tunnel of SMDM project (Fig. 3b), the target variable PRnet is negatively correlated with input features such as CRS, torque, thrust, and RMR with the values of −0.05, −0.47, −0.59, and − 0.36, respectively. On the other hand, it is positively correlated with PRchd, weathering grade, and rock strength, with correlation coefficients of 0.89, 0.26, and 0.13, respectively. The low correlation values do not indicate multicollinearity, which in fact can help improve model performance by reducing overfitting^30^. Katuwal and Panthi (2025)^33^ highlight that Rock strength and weathering grade provide a valuable understanding for evaluating TBM performance in TBM jamming or stuck areas. Therefore, all parameters were selected as input features. The statistical description of selected input and target variables for both project database is presented in Table 2.

**Table 2:** Statistical description of selected input features and target variable.

Project	Parameters	Count	Mean	Std.	Min.	25%	50%	75%	Max.
BBDM project	CRS (rpm)	8614	7.50	0.92	2.60	7.00	8.00	8.00	9.50
	PRchd (mm/rev)	8614	9.13	2.11	1.70	7.60	9.00	10.50	17.10
	Torque (kNm)	8614	321.14	58.98	142	276	320	360	546
	Thrust (kN)	8614	5020.10	1047.96	2059	4265.5	5000	5783	11,800
	RMR	8614	38.48	7.29	14	35	38	42	60
	PRnet (mm/min)	8614	67.77	15.53	13	56	66	79.1	128.5
SMDM project	CRS (rpm)	9470	6.72	0.61	2.50	6.50	7.00	7.00	9.00
	PRchd (mm/rev)	9470	8.59	2.47	1.60	7.10	8.30	9.90	18.80
	Torque (kNm)	9470	642.35	284.81	82	387	662	890	2900
	Thrust (kN)	9470	6835.47	2138.42	3067	5326	6263	7837	28,300
	RMR	9470	39.76	9.92	16	35	39	46	60
	PRnet (mm/min)	9470	57.03	15.20	9.60	48.20	56.20	64.20	99.70

As seen in Table 2, the mean values of key rock-breaking parameters vary significantly across the projects. This variability is primarily due to differences in TBM machine features and geological conditions across the projects. In the headrace tunnel of SMDM project, the mean values of the response parameters, i.e., torque and thrust, are approximately 2 and 1.36 times higher than those in the headrace tunnel of BBDM project. In contrast, the control parameters, such as CRS and PRchd, have slightly lower mean values in the headrace tunnel of SMDM project, which are about 0.90 and 0.94 times those observed in the headrace tunnel of BBDM project. Despite these differences, the average PRnet remains relatively consistent across both projects, suggesting that the overall penetration performance of the TBM machines is comparable.

### Data distributions and standardization

**Fig. 4:**
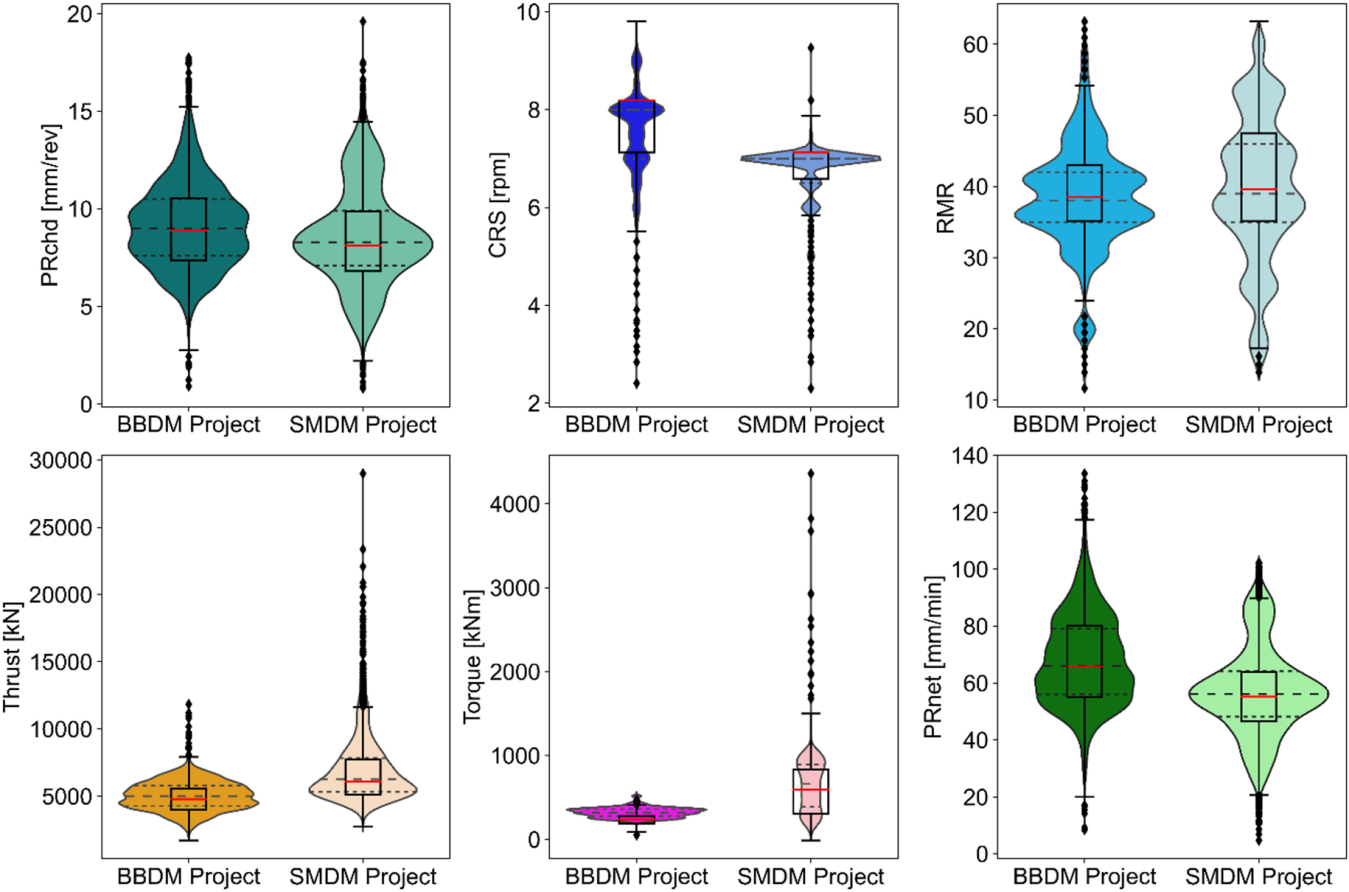
Cross-project data distribution comparison using a combination of violin and box plot, where CRS: Cutterhead speed, PRchd: Cutterhead penetration rate, RMR: Rock mass rating, and PRnet: Net penetration rate.

Understanding the distribution of data across different projects is crucial for visualizing the range and variability of selected features in relation to the respective projects. Figure 4 presents a combined box-and-violin plot that captures detailed distribution of cross-project data. This visualization highlights key statistical characteristics of each variable, including central tendency, spread, outliers, distribution shape, and density.

As illustrated in Fig. 4, the distributions of PRchd, CRS, and rock mass quality are relatively consistent across both projects. In contrast, the distributions of thrust and torque exhibit considerable variability, primarily due to the presence of potential outliers, which are marked as black dots in the plots. These outliers are likely linked to the complex and challenging geological conditions encountered along the headrace tunnel alignments in both projects. It is important to note that these outliers offer valuable insights into the geological conditions and the corresponding TBM performance^7,33^.

The selected input and target variables display differing ranges in their data distributions (Table 2). Additionally, the shapes of the violin density plots reveal that most features deviate from a normal (Gaussian) distribution (Fig. 4). Hence, the selection of an appropriate scaling technique becomes essential to ensure consistency across feature magnitudes and robust model performance. Several scaling approaches are commonly used, including normalization, standardization, and robust scaling. Normalization relies on the minimum and maximum values of each feature and is therefore highly sensitive to outliers. Standardization uses the mean and standard deviation, making it suitable for features that follow roughly Gaussian distributions; however, it remains moderately sensitive to outliers. In contrast, robust scaling uses the median and interquartile range (IQR), which makes it effective for features containing outliers and those that do not follow normal distributions^35^. Given that the features in this study show clear non-normal distribution and the presence of outliers in the data, a robust scaling method was selected to scale the dataset effectively. Following to Brownlee (2020)^35^, Eq. 1 was employed to scale the selected features.

1 $$\:{\mathrm{X}}_{\mathrm{r}\mathrm{o}\mathrm{b}\mathrm{u}\mathrm{s}\mathrm{t}}=\:\frac{\mathrm{x}-\mathrm{m}\mathrm{e}\mathrm{d}\mathrm{i}\mathrm{a}\mathrm{n}}{{\left({\mathrm{Q}}_{3}\right)}_{\mathrm{x}}-{\left({\mathrm{Q}}_{1}\right)}_{\mathrm{x}}}$$

where x denotes the input feature values, and Q_1_​ and Q_3​_ represent the 25th and 75th percentiles, respectively.

**Fig. 5:**
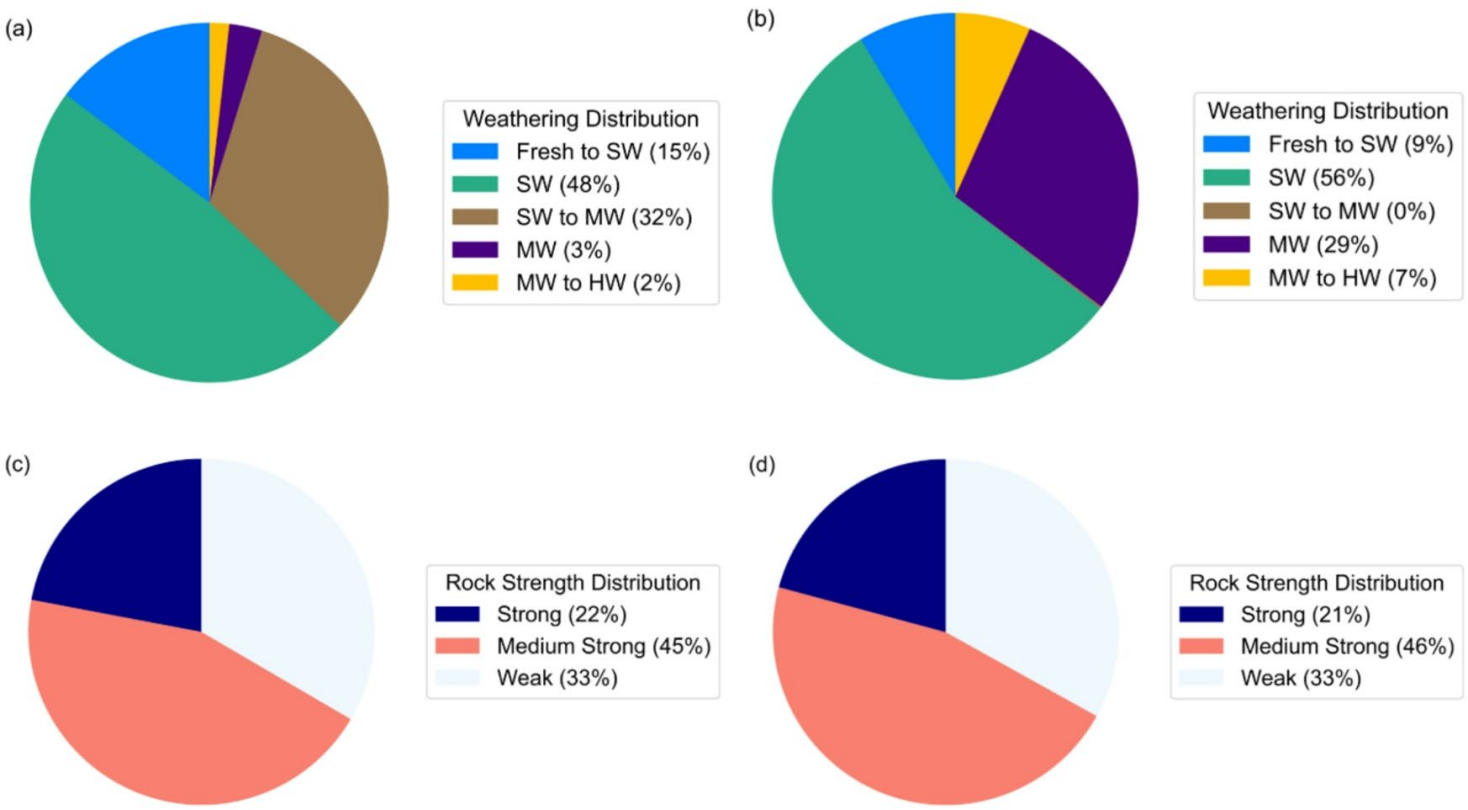
Rock weathering and strength condition distribution; (**a**) BBDM project, (**b**) SMDM project, (**c**) BBDM project, (**d**) SMDM project.

In these projects, weathering and rock strength conditions were classified following guidelines given by International Society of Rock Mechanics and Rock Engineering (ISRM). Following guidelines ISRM, (1978)^36^, the rock mass weathering conditions along the headrace tunnel have been classified as fresh to slightly weathered (Fresh to SW), slightly weathered (SW), slightly to moderately weathered (SW to MW), moderately weathered (MW), and moderately to highly weathered (MW to HW). As seen in Figs. 5a and 48% of the rock mass at BBDM headrace tunnel belongs to SW condition, followed by SW to MW, Fresh to SW, MW, and MW to HW consisting of 32%, 15%, 3%, and 2%, respectively. Figure 5b shows the weathering condition at SMDM where 56% rock mass belongs to SW condition, followed by MW, Fresh to SW, and MW to HW consisting of 29%, 9%, and 7%, respectively.

Rock strength along the headrace tunnel was assessed based on the ISRM (1979)^37^ classification system, which defines seven strength categories, i.e., extremely weak (0.25–1 MPa), very weak (1–5 MPa), weak (5–25 MPa), medium strong (25–50 MPa), strong (50–100 MPa), very strong (100–250 MPa), and extremely strong (> 250 MPa). Following this classification, the rock mass strength in these two headrace tunnels belongs to strong, medium-strong, and weak (Fig. 5c, d).

### Model selection and hyperparameter tuning

In this study, ensemble methods that include bagging^38,39^, random forest (RF)^40^, boosting/eXtreme Gradient Boosting^39,41,42^, stacking ensemble^43,44^ and artificial neural network^44–46^ were employed. The models have been developed using the scikit-learn, XGBoost, and TensorFlow (Keras) libraries in Python. Each of these algorithms offers distinct advantages, and a performance comparison among these helps to establish a robust predictive model while avoiding reliance on a single algorithm and minimizing the risk of overfitting.

Hyperparameter tuning is a crucial step in ML prior to the actual training process. It reduces overfitting and underfitting, enhances model generalization, and improves overall performance^14^. In this study, hyperparameter tuning for each ensemble model was performed using the GridSearch approach with a fixed 5-fold configuration. The models were trained over a range of hyperparameters, including combinations of default parameters provided by the respective Python libraries. On the other hand, the Hyperband algorithm from the Keras Tuner library was used to optimize hyperparameters for the artificial neural network (ANN) model. In the ANN model, a batch size of 32, optimal epoch of 36, and adaptive moment estimation (Adam) algorithm were employed to enhance the optimization process. The details of selected optimal hyperparameters are presented in Table 3.

**Table 3:** Selected optimal hyperparameters for different ML models.

Regression model	Hyperparameters	Search space	Optimized hyperparameters
1. Bagging	n_estimators	10–300, step = 10	100
	max_samples	0.1–1.1, step = 0.1	0.5
	max_features	0.1–1.1, step = 0.1	1
2. RF	n_estimators	10–300, step = 10	200
	max_depth	5–50, step = 5	30
	max_features	‘sqrt’, ‘log2’	‘log2’
	min_samples_leaf	1–10, step = 1	1
	min_samples_split	1–10, step = 1	5
3. XGBoost	n_estimators	10–300, step = 10	100
	max_depth	1–10, step = 1	7
	learning_rate	0.01–0.3, setp = 0.05	0.1
	gamma	0–10, step = 1	5
	subsample	0.1–1.0.1.0, step = 0.1	0.8
	colsample_bytree	0.1–1.0.1.0, step = 0.1	1
4. ANN			
Hidden layer	Hidden layer number	1–5	1
	Neurons per layer	32–512, step = 32	352
	Activation function	‘ReLU’, ‘tanh’, ‘sigmoid’, ‘linear’	‘ReLU’
	Kernel initializer	‘uniform’, ‘glorot_uniform’, ‘he_normal’	‘glorot_uniform’
Output layer	Kernel initializer	‘uniform’, ‘glorot_uniform’, ‘he_normal’	‘he_normal’
	Activation function	‘linear’	‘linear’

### Cross-project model development and evaluation

The predictive performance of the developed models was initially evaluated using the BBDM project database, which was split into training and testing sets following an 80/20 rule. Subsequently, a cross-project evaluation framework was implemented to assess model generalizability. For this purpose, datasets from both TBM projects were combined to form a comprehensive database. The selected models were trained and tested under different scenarios, and their performance was evaluated using the coefficient of determination (R²) and standard loss functions (error metrics), including mean absolute error (MAE), root mean squared error (RMSE), and mean absolute percentage error (MAPE). The different training and testing scenarios are defined as follows:

Scenario 1: Train on BBDM project and Test on SMDM project (Siwalik database).

Scenario 2: Train on combined dataset from BBDM and SMDM projects (Siwalik database) and Test on SMDM project Lesser Himalayan database.

Scenario 3: Train and test on a combined stratified dataset from BBDMP and SMDMP using 5-fold cross-validation.

In Scenario 3, datasets from both BBDM and SMDM projects were combined into a single database, and a project identifier was assigned to each sample. To maintain data stratification, the combined dataset were divided into five equal subsets, considering the project identifier and the distribution of geological conditions (Fig. 5). The approach minimizes bias arising from class imbalance and allows for a systematic evaluation of model generalizability. These five subsets were subsequently utilized in five-fold cross-validation with an 80/20 train/test splits. Model performance across the five folds was further evaluated using standard metrics along with 95% confidence interval (CI) analysis.

## Results and discussion on prediction model

### Model on BBDM project database

The regression models were trained and tested on the BBDM project dataset using an 80/20 split. The predictive performance of these models is summarized in Table 4. The evaluation metrics indicate that the R² values on the training set range from 0.957 to 0.980 and on the testing set range from 0.936 to 0.938, respectively, demonstrating high prediction accuracy.

**Table 4:** Prediction model performance in a single project (BBDM project) database.

Model	Training Set				Testing Set			
	*R* ^2^	MAE	RMSE	MAPE	*R* ^2^	MAE	RMSE	MAPE
XGBoost	0.957	2.018	4.174	3.039	0.936	2.187	3.996	3.410
RF	0.980	1.292	2.196	2.000	0.936	2.397	3.981	3.743
Bagging	0.971	1.501	2.651	2.303	0.936	2.331	3.968	3.634
Stacking	0.962	1.654	2.610	2.477	0.938	2.398	3.929	3.753
ANN	0.964	1.475	2.494	2.239	0.938	2.321	3.925	3.587

As presented in Table 4, all ensemble and ANN models exhibit superior performance. However, these model training and validation processes were performed using data with similar TBM features and geological conditions from the Siwalik region of the Nepal Himalaya. To assess their robustness and practical applicability, it is crucial to evaluate their prediction performance on tunnelling datasets from other projects characterized by different geological conditions and TBM configurations. To address this, three cross-project scenarios (as discussed in Sect. 2.5) were implemented.

### Scenario 1

In this scenario, all data from the BBDM project were used to train the models with the corresponding optimized hyperparameters. The trained models were then tested on an independent and unseen SMDM project dataset from a similar Siwalik geological region, although the two projects used slightly different TBM features. The performance metrics for these models are summarized in Table 5.

**Table 5:** Prediction model performance on cross–project database (Scenario 1).

Model	Training Set				Testing Set			
	*R* ^2^	MAE	RMSE	MAPE	*R* ^2^	MAE	RMSE	MAPE
XGBoost	0.969	1.707	2.734	2.570	0.838	2.384	3.271	4.720
RF	0.979	1.361	2.261	2.110	0.416	5.011	6.220	9.260
Bagging	0.972	1.468	2.578	2.260	0.823	2.567	3.422	4.990
Stacking	0.950	2.015	3.346	3.100	0.749	3.131	4.077	5.980
ANN	0.914	2.228	4.541	3.430	0.606	3.521	5.110	7.370

The results shown in Table 5 indicate that the R² values on the training set range between 0.914 and 0.979 for Scenario 1. On the other hand, the R² values on the testing set vary widely, ranging from 0.416 to 0.838. While the training performance remains consistent, the prediction accuracy is found to be notably lower compared to that observed within the same project data. This is specifically the case for testing sets for the RF and ANN regression models, which exhibit relatively low R² values of 0.416 and 0.606, respectively. Meanwhile, the stacking, bagging, and XGBoost models performed relatively better, achieving fairly good R² values of 0.749, 0.823, and 0.838, respectively. These findings indicate that the predictive performance of RF and ANN models is highly sensitive to variations in geological conditions and machine parameters. Both models tend to overfit the training project data, capturing site-specific patterns that do not generalize well to other TBM projects, resulting in poor cross-project performance compared to other models. Hence, the prediction performance of the model analyzed with Scenario 1 indicates the need to include a diverse database to enhance reliability of the model.

### Scenario 2

In Scenario 2, the Siwalik region datasets from the BBDM and SMDM projects were merged to create a combined dataset, which was then used to train the selected ML models. The trained models were subsequently tested on an independent, unseen dataset from the headrace tunnel of the Lesser Himalayan region in the SMDM project. The performance metrics for these models are summarized in Table 6.

**Table 6:** Prediction model performance on cross–project database (Scenario 2).

Model	Training Set				Testing Set			
	*R* ^2^	MAE	RMSE	MAPE	*R* ^2^	MAE	RMSE	MAPE
XGBoost	0.974	1.426	2.424	2.220	0.972	1.708	2.919	3.330
RF	0.983	1.156	2.00	1.860	0.895	3.569	5.683	6.400
Bagging	0.978	1.189	2.252	1.870	0.986	1.288	2.108	2.210
Stacking	0.961	1.648	2.995	2.600	0.978	1.585	2.610	2.930
ANN	0.982	1.233	2.341	3.160	0.933	1.755	3.928	2.750

The results in Table 6 show that the R^2^ values on the training set range between 0.961 and 0.983, while the R^2^ values on the testing set range between 0.895 and 0.986. The results indicate that almost all models have demonstrated better performance than that in Scenario 1. These findings suggest that incorporating a broader range of TBM operational parameters in the ML process enhances model generalization, thereby improving prediction performance on unseen and diverse datasets.

### Scenario 3

Since the merging strategy adopted in Scenario 2 demonstrated better performance, its application in Scenario 3 is expected to further enhance model accuracy. Therefore, datasets from both the BBDM and SMDM projects were combined using stratification to create a more diverse and representative dataset. This combined dataset encompasses varied operational conditions from both the Siwalik and Lesser Himalayan regions, as well as a wider range of geological and machine parameters.

**Fig. 6:**
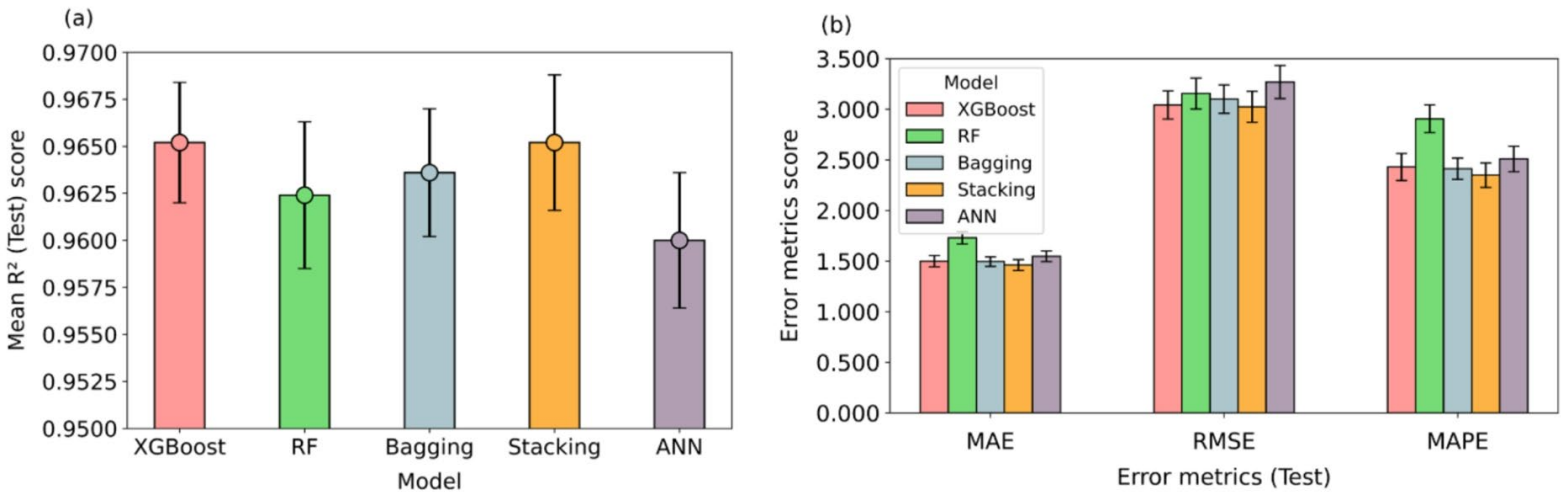
Model performance comparison with 95% CI across five folds in Scenario 3: (**a**) R2 values; (**b**) Error metrics.

Following the cross-validation strategy described in Sect. 2.5 (Scenario 3), the five-fold mean R^2^ and error metric values with corresponding 95% CI for the various ML models are presented in Fig. 6a, b. The mean R^2^ value of each model is represented by a circular marker at the top of the vertical bar. The colored bars indicate the 95% confidence intervals of the performance metrics for XGBoost (red), RF (green), bagging (blue), stacking (orange), and ANN (purple). Vertical black lines denote the corresponding error bars for each model.

All selected models show comparable R^2^ values having greater than 0.960 (Fig. 6a). The margin of error ranges from ± 0.0032 to ± 0.0039, which indicates a very narrow interval. This demonstrates that the models generalize well across folds with highly consistent performance. As reported by Timilsina et al.^47^, a narrow margin of error indicates low variance in model performance across repeated experiments. The results further confirm that the observed model performance is statistically very good (p-value < 0.001).

As seen in Fig. 6a, XGBoost and stacking achieved the highest mean R^2^ values of 0.965, while ANN showed the lowest value of 0.960. Notably, the 95% CI for XGBoost across the five folds is narrower than that of the stacking model. Therefore, despite comparable mean R^2^ values, a narrower CI reflects more balanced and robust performance. The error metrics of the selected ML models are presented in Fig. 6b. XGBoost and stacking models exhibit the lowest and comparable MAE, RMSE, and MAPE values. The RF model shows higher MAE and MAPE, while the ANN model exhibits a higher RMSE. Overall, the findings indicate that the model reliably captures the relationship between input features and TBM PRnet. The adopted approach ensures robust predictions under diverse geological and operational conditions. Similar performance trends were observed across all selected models (Table 7).

**Table 7:** Prediction model performance on cross–project database (Scenario 3).

Model	Training Set				Testing Set			
	*R* ^2^	MAE	RMSE	MAPE	*R* ^2^	MAE	RMSE	MAPE
XGBoost	0.984	1.132	2.042	1.814	0.965	1.499	3.042	2.430
RF	0.989	0.947	1.715	1.582	0.962	1.730	3.155	2.906
Bagging	0.987	0.910	1.878	1.470	0.964	1.495	3.101	2.413
Stacking	0.986	0.983	1.922	1.573	0.965	1.464	3.021	2.351
ANN	0.960	1.538	3.258	2.488	0.960	1.547	3.269	2.508

The results summarized in Table 7 show high predictive accuracy with lower loss functions for Scenario 3. The R^2^ values on the training sets range from 0.960 to 0.989, while on the test sets range from 0.960 to 0.965. Among the evaluated models, XGBoost and stacking have achieved highest R^2^ value of 0.965, while ANN model showed the lowest value of 0.960. Despite this small variation, all models have demonstrated strong prediction capabilities with R^2^ values exceeding 0.960, which confirms their robustness in predicting PRnet.

In summary, all selected models demonstrate good performance. Among these, the XGBoost and stacking models show highest performance. Notably, the XGBoost model exhibits a lower margin of error compared to the stacking model, despite comparable error metrics. Both models are suitable for further prediction; however, in this study, XGBoost was selected for subsequent analysis. Overall, the analysis shows that the use of combined stratified and cross-validation enabled the models to effectively capture the complexity and diversity of geological and TBM operation parameters.

### SHAP-based interpretability analysis

**Fig. 7:**
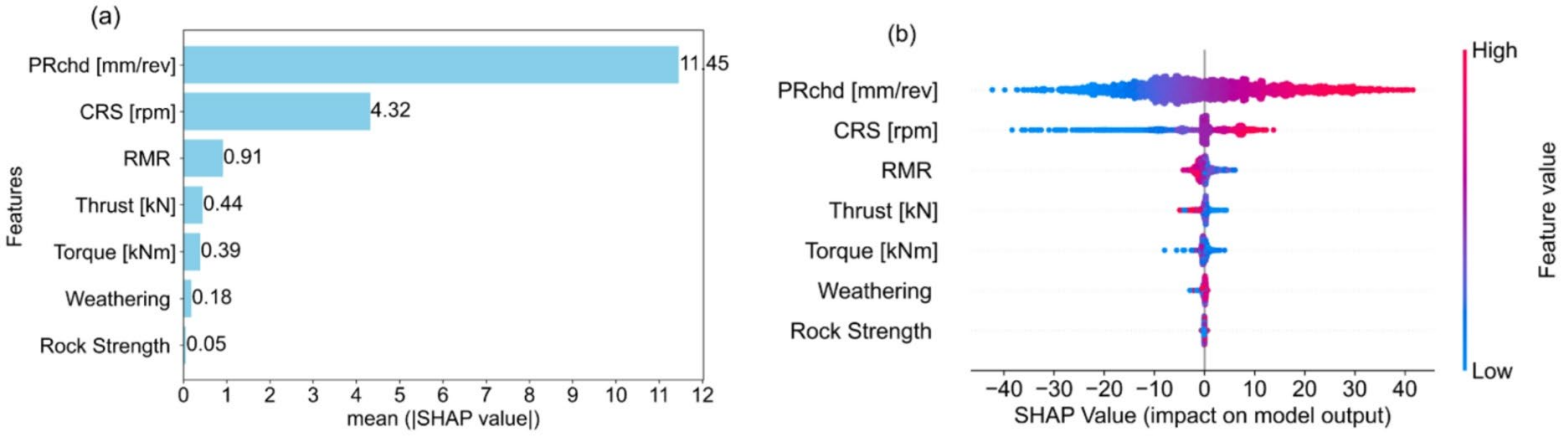
(**a**) Features importance analysis using mean absolute SHAP values for each variable, (**b**) Beeswarm plot ranked by mean SHAP value.

Model transparency is essential for quantifying the contribution of individual input features to an ML model predictions. SHAP is an interpretability framework grounded in cooperative game theory. In this study, SHAP was employed to evaluate the relative importance and influence of the selected input features on the TBM PRnet. The global interpretation results generated using SHAP for the best-performing XGBoost model are presented in Fig. 7.

The mean absolute SHAP value for each input feature across the entire database indicates its average contribution to the TBM PRnet (Fig. 7a). As seen in the figure, the mean SHAP values display descending order of importance following their relative magnitudes. In Fig. 7a, PRchd is the most influential variable, contributing on average 11.45 mm/min to the TBM PRnet. In contrast, rock strength is the least influential variable, with an average contribution of only 0.05 mm/min. The other parameters such as CRS, RMR, torque, thrust, and weathering show average contribution values of 4.32, 0.91, 0.44, 0.39, and 0.18 mm/min, respectively.

The SHAP beewarm plot ranks the input features in descending order from top to bottom based on mean SHAP values for entire database (Fig. 7b). In the figure, the horizontal axis illustrates influence of each feature on the model’s prediction. The SHAP values of individual data points are distributed horizontally for each input feature. Data points on the right indicate positive SHAP values, meaning the feature increases the TBM PRnet, while data points on the left indicate negative SHAP values, meaning the feature decreases it. In addition, blue and red colors represent low and high feature values, respectively. Vertically stacked points reflect a higher density of SHAP values, highlighting regions where many observations have similar contributions.

As seen in Fig. 7b, the results show that PRchd has strong positive effect on PRnet compared to CRS and RMR. Among the response variables, thrust and torque exhibit a negative influence on the PRnet prediction. Weathering and rock strength demonstrate a neutral to slightly positive effect on PRnet.

**Fig. 8:**
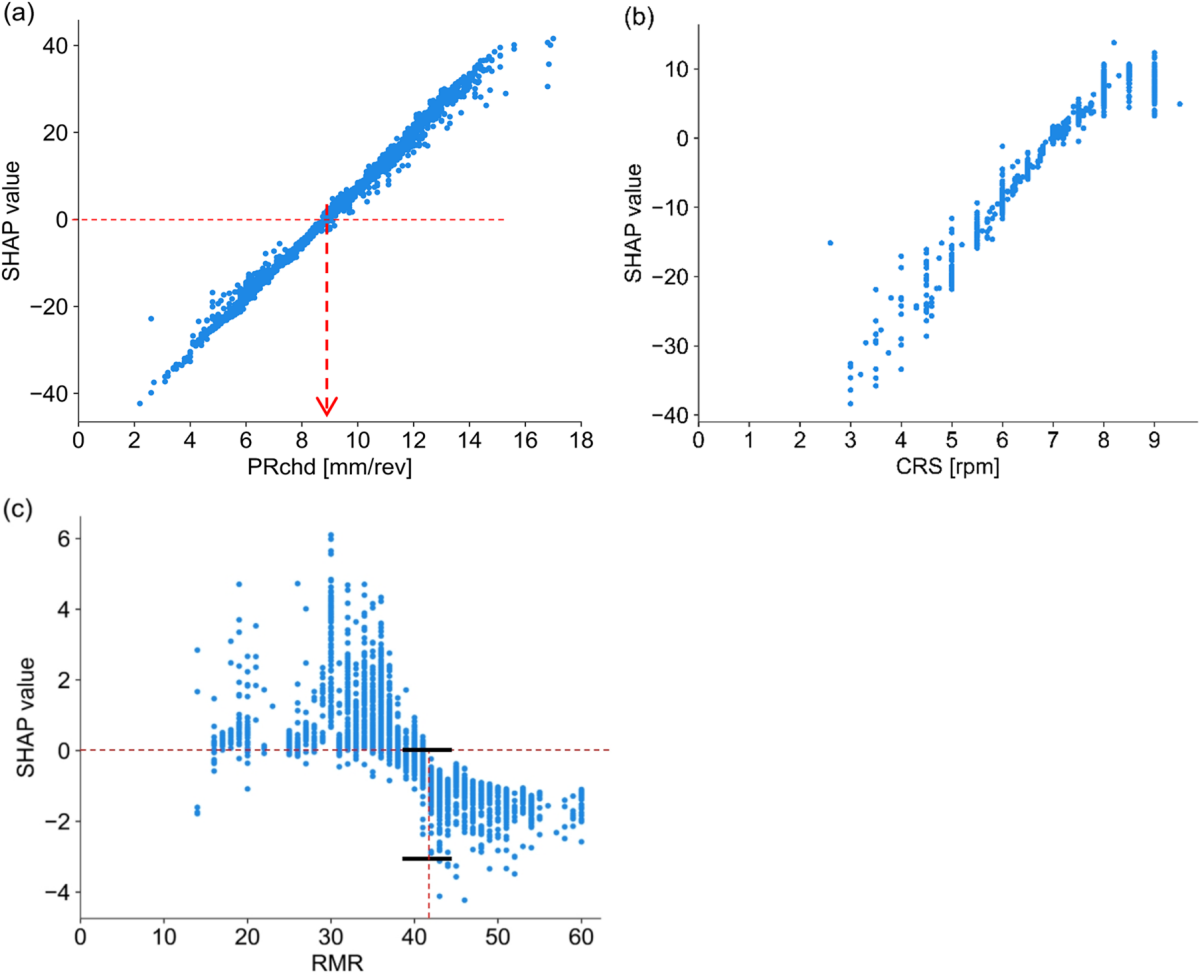
Dependence plots for top three important features; (**a**) PRchd, (**b**) CRS, (**c**) RMR.

In this study, dependence plots were employed to evaluate the effect of individual input features across the dataset. These plots illustrate the relationship between feature values and the model’s predicted outputs. The top three features consisting of PRchd, CRS, and RMR were selected to analyze their effect on the TBM PRnet (Fig. 8a, b,c). The dependence plot of each displays original values on the x-axis and corresponding SHAP values on the y-axis. The relationship between SHAP values and original values differs across features. As seen in Fig. 8a, b, PRchd and CRS exhibit a clear positive trend having approximately linear distribution with SHAP value ranges. For RMR values up to 40 show positive SHAP values, whereas RMR values higher than 40 show a negative trend. This suggests that RMR values up to about 40 improve the TBM PRnet, whereas higher values tend to reduce it.

SHAP values above the horizontal reference line (y = 0) contribute positively to PRnet, whereas values below this line influence negatively. For example, a PRchd value of around 9 mm/rev marks the transition from negative to positive contribution, shifting the model’s prediction toward higher PRnet (Fig. 8a). The vertical spread of SHAP values in each plot reflects the influence of interactions with other features. PRchd exhibits wider SHAP value ranges, followed by CRS and RMR. For example, in Fig. 8c, RMR value of 42 produces SHAP values ranging from 0 to – 3 mm/min, depending on interactions with other feature values associated with those observations.

### TBM jamming risk assessment

In TBM excavation, unexpected jamming and TBM stuck events are among the most critical issues encountered when tunnel passes through weak rocks and fault zones. Jamming and TBM stuck not only reduce the excavation progress but also increase project costs and time of completion. As described earlier, the TBM response parameters such as torque, thrust, and corresponding rock mass conditions influence the TBM PRnet. The associated parameters from cross-project database were selected to assess the potential risk of TBM jamming.

### TBM parameter behaviour assessment

**Fig. 9:**
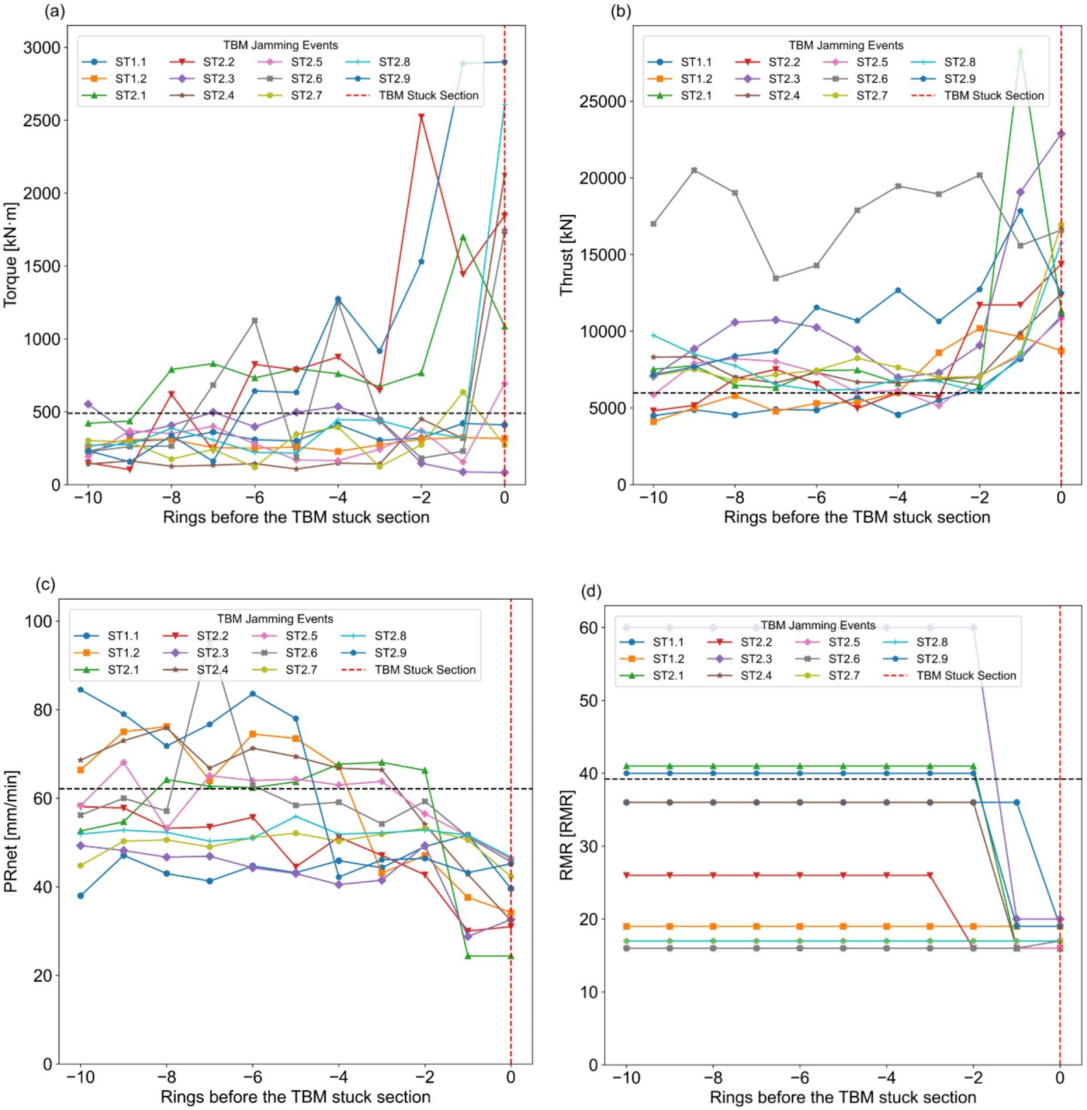
Trends in TBM parameter fluctuations over the 10 Rings before the stuck section: (**a**) Torque, (**b**) Thrust, (**c**) PRnet, (**d**) RMR.

TBM jammed events from both BBDM and SMDM projects were evaluated using TBM parameters of ten rings before each stuck section. The trend of all TBM jamming and stuck events for both projects is presented in Fig. 9a, b,c, d. At BBDM project, the TBM jammed at two locations, which are designated as ST1.1 and ST1.2. Similarly, at SMDM project, the TBM jammed at nine different locations, which are labeled from ST2.1 to ST2.9 in Fig. 9. The black dotted line in Fig. 9 represents the mean value of respective TBM parameter, which can be used as a reference for comparative assessment.

As seen in Fig. 9a, torque values fluctuate noticeably when approaching TBM jamming section. In most of the jamming cases, a sharp increase in torque is observed at one or two rings before hitting the jamming section. All jamming events exhibited significantly higher peak torque values compared to the mean value of combined database. The jamming events ST1.1 and ST1.2 generally follow this pattern, although their torque magnitudes remain below the mean value. In contrast, the events ST2.3 and ST2.7 do not obey this trend. Figure 9b illustrates behavior thrust while approaching the jamming section. Similar to torque, a steep increase in thrust at two rings before the jamming section with peak thrust values occurring at jamming events and exceeding the mean thrust of the combined dataset is observed.

As seen in Fig. 9c, the trend of PRnet exhibits significant fluctuations near the jamming section, where a sudden drop below the mean of combined database occurs at two rings before jamming. Figure 9d highlights surrounding rock mass quality conditions at ten rings before the TBM jamming section. The field mapping results indicate that the rock mass quality suddenly dropped from fair rock mass class (class III) or poor rock mass class (class IV) to very poor rock mass class (class V) at one or two rings before the jamming section. Subsequently, the TBM jammed at tunnel section where very poor rock mass conditions exist.

### Prediction of potential jamming events

As discussed earlier, TBM operational parameters show high fluctuations under class V rock mass conditions, especially when approaching sections prone to TBM jamming. These parameters tend to spike sharply at stuck sections. As reported by Katuwal and Panthi^33^, lower PRnet values (below 25th percentile) combined with large fluctuations in torque and thrust (exceeding 75th percentile) serve as strong indicators of potential challenges, such as TBM getting stuck or the cutterhead becoming jammed.

**Fig. 10:**
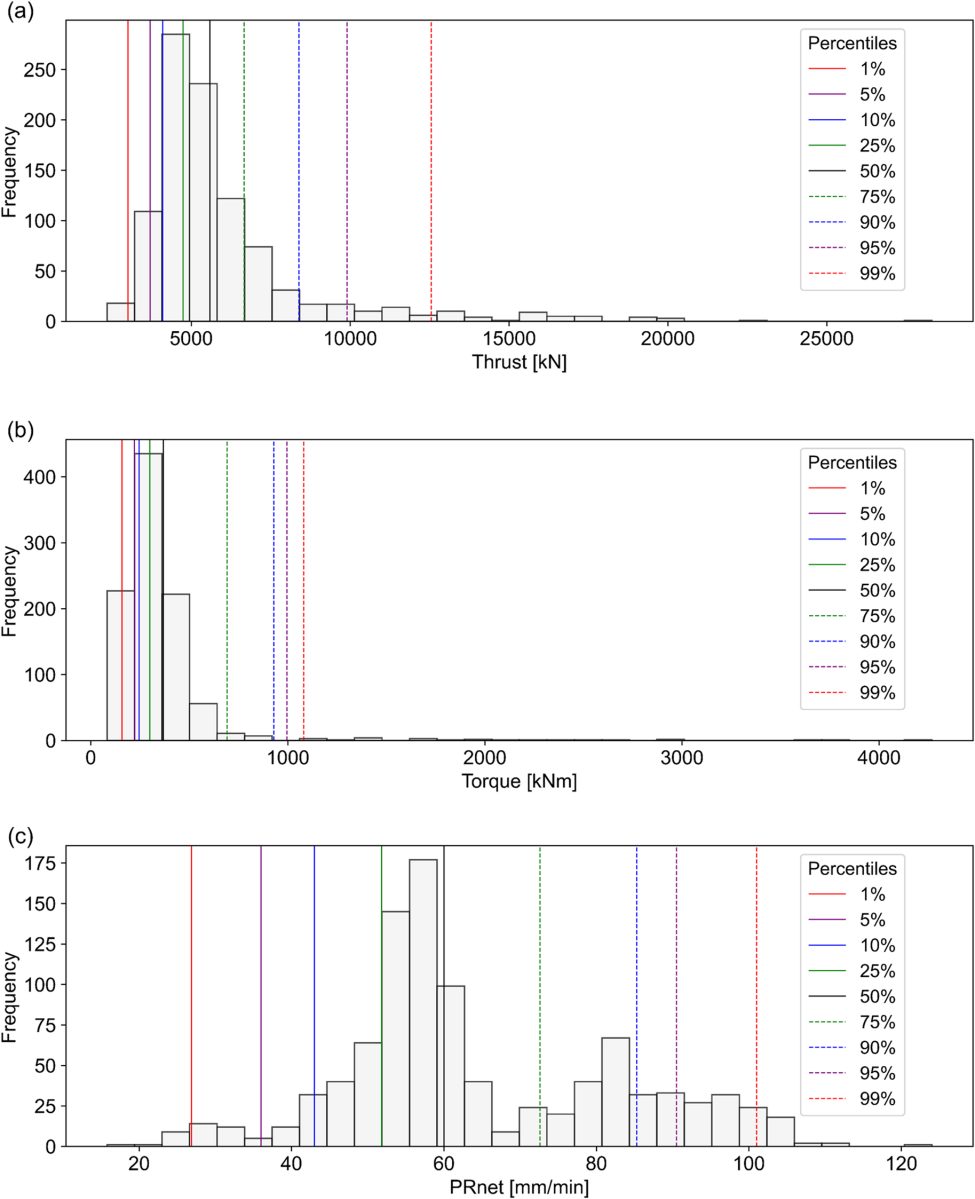
Statistical distribution of TBM parameters in class V: (a) Torque, (b) Thrust, (c) PRnet.

To assess these variability patterns in greater detail, the statistical distributions of torque, thrust, and PRnet for class V conditions are presented in Fig. 10. The vertical axis represents the frequency of occurrence, while the horizontal axis shows the parameter range within class V. Percentile lines (P_1_, P_5_, P_10_, P_25_, P_50_, P_75_, P_90_, P_95_, and P_99_) are presented on the histograms using different colors and line styles. These percentiles help visualize the variability characteristics of each parameter and provide practical cutoff thresholds for assessing potential jamming risks. Noticeable changes in the behavior of torque, thrust, and PRnet can be observed across these percentile intervals (Fig. 10). For example, the lower PRnet values in class V (Fig. 10c) appear to serve as indicators of TBM jamming risk, consistent with the findings presented earlier in Sect. 2.3 (Fig. 4). Based on these results, PRnet values below the P_5_ are classified as highly variable. Values between P_5_ and P_25_ are categorized as moderately variable, and those between P_25_ and the mean are considered slightly variable. PRnet values exceeding the mean reflect relatively better TBM performance under class V conditions and are categorized as normal. This percentile-based threshold system is applied to evaluate PRnet variability within class V rock mass condition.

Further, Katuwal and Panthi (2025)³⁴ reported that thrust and torque requirements are generally lower in class V compared to class IV and III. However, this analysis showed thrust and torque exhibiting high variability beyond the P_95_, which appears contradictory to typical expectations. Similar observations are also discussed in Sect. 2.3 (Fig. 4). Based on these results, a percentile-based variability scoring system was developed to classify the probability of potential jamming during TBM tunnelling. The assigned variability thresholds for torque, thrust, and PRnet, along with the corresponding variable classes and scores, are presented in Table 8.

**Table 8:** Thresholds for variability classification and associated scoring system.

Variability Threshold		Variability Classifications	
Torque/Thrust	PRnet	Variable Class	Score
value > P_95_	value < P_5_	Highly variable (HV)	3
P_75_ < value ≤ P_95_	P_5_ ≤ value < P_25_	Moderately variable (MV)	2
mean < value ≤ P_75_	P_25_ ≤ value < mean	Slightly variable (SV)	1
all others	all others	Normal	0

In this study, the variability in TBM parameters is categorized into four classes: highly variable, moderately variable, slightly variable, and normal, as summarized in Table 8. This scoring system was used to analyze the variability conditions along the actual TBM jamming or stuck sections. The resulting classifications are presented in Table 9.

**Table 9:** TBM parameter variability conditions in very poor rock mass class (class V).

TBM Stuck Sections	Torque variability			Thrust variability			PRnet variability		
	−2 ring	−1 ring	0 ring	−2 ring	−1 ring	0 ring	−2 ring	−1 ring	0 ring
ST1.1	N/A	N/A	Normal	N/A	N/A	HV	N/A	N/A	MV
ST1.2	Normal	Normal	Normal	MV	HV	HV	MV	HV	HV
ST2.1	N/A	HV	HV	N/A	HV	HV	N/A	HV	HV
ST2.2	HV	HV	HV	HV	HV	HV	MV	HV	HV
ST2.3	N/A	Normal	Normal	N/A	HV	HV	N/A	HV	HV
ST2.4	N/A	Normal	HV	N/A	HV	HV	N/A	MV	HV
ST2.5	Normal	Normal	MV	MV	HV	HV	SV	MV	MV
ST2.6	Normal	Normal	HV	HV	HV	HV	SV	MV	MV
ST2.7	Normal	SV	Normal	MV	HV	HV	SV	MV	MV
ST2.8	Normal	Normal	HV	SV	HV	HV	SV	MV	MV
ST2.9	N/A	HV	HV	N/A	HV	HV	N/A	MV	MV

As shown in Table 9, the torque values exhibit variability ranging from normal to high. In some tunnel sections, variations in torque fall within the normal or slightly variable class indicating no TBM jamming. However, inconsistent torque results at actual TBM jamming sections represent difficulties in jamming risk evaluation. Therefore, torque parameter is excluded from jamming risk prediction model. On the other hand, the thrust and PRnet parameters consistently exhibited high variability across all jamming sections. Thrust values with high variability and PRnet values ranging from moderate to high variability one ring prior to jamming section are indicative of risk levels. In some jamming cases, such as ST1.2 and ST2.2, moderate to high variability is observed at two rings before the stuck sections. Similarly, for ST1.1, moderate to high variability is observed only at the jamming section itself, which can be attributed to a change in rock mass quality conditions from class III or IV to class V for the particular rings, denoted as not applicable (N/A). The finding indicates that variations in thrust and PRnet in the ring before reaching to jamming section can serve as reliable predictors of impending TBM jamming. Hence, a combined jamming risk (CJR) score is proposed to classify the risk level into four categories: high risk, medium risk, low risk, and no risk. The corresponding CJR score values for each risk category are summarized in Table 10.

**Table 10:** Risk level classification under very poor rock conditions.

Combined jamming risk (CJR) score	Risk level class
5–6	High risk
3–4	Medium risk
1–2	Low risk
0	No risk

The proposed CJR score risk assessment was applied to validate predictive performance on actual TBM jamming sections (Table 11). The CJR scoring system demonstrated fairly reliable performance by flagging a high-risk warning at least one ring prior to TBM jamming event. Table 11 indicates that, in addition to the known jamming sections, several other tunnel sections have potential risk for TBM jamming showing high-risk flags up to three rings before jamming event.

**Table 11:** CJR score based on risk level classification.

Project	Chainage (m)	Ring No.	Potential Jamming Indication	Risk Level	Remarks
BBDM	8588.5	6028	0 ring	High Risk	ST1.1
	8606.7	6041	1 ring before	High Risk	ST1.2
SMDM	2156.678	1503	1 ring before and after	High Risk	ST2.1
	4669.332	3286	2 rings before and 9 rings after	High Risk	ST2.2
	9226.985	6536	1 ring before and 5 rings after	High Risk	ST2.3
	9252.520	6554	4 rings before	High Risk	Potential Jamming Zone
	10488.023	7434	1 ring before	High Risk	ST2.4
	10512.062	7451	1 ring before	High Risk	ST2.5
	10528.019	7461	4 rings before	High Risk	Potential Jamming Zone
	10544.858	7473	1 ring before	High Risk	ST2.6
	10569.226	7492	1 ring before	High Risk	ST2.7
	10583.808	7503	1 ring before and 2 rings after	High Risk	ST2.8
	10602.008	7516	1 ring before	High Risk	Potential Jamming Zone
	12619.780	8953	1 ring before	High Risk	ST2.9
	12823.040	9103	3 rings before	High Risk	Potential Jamming Zone

**Fig. 11:**
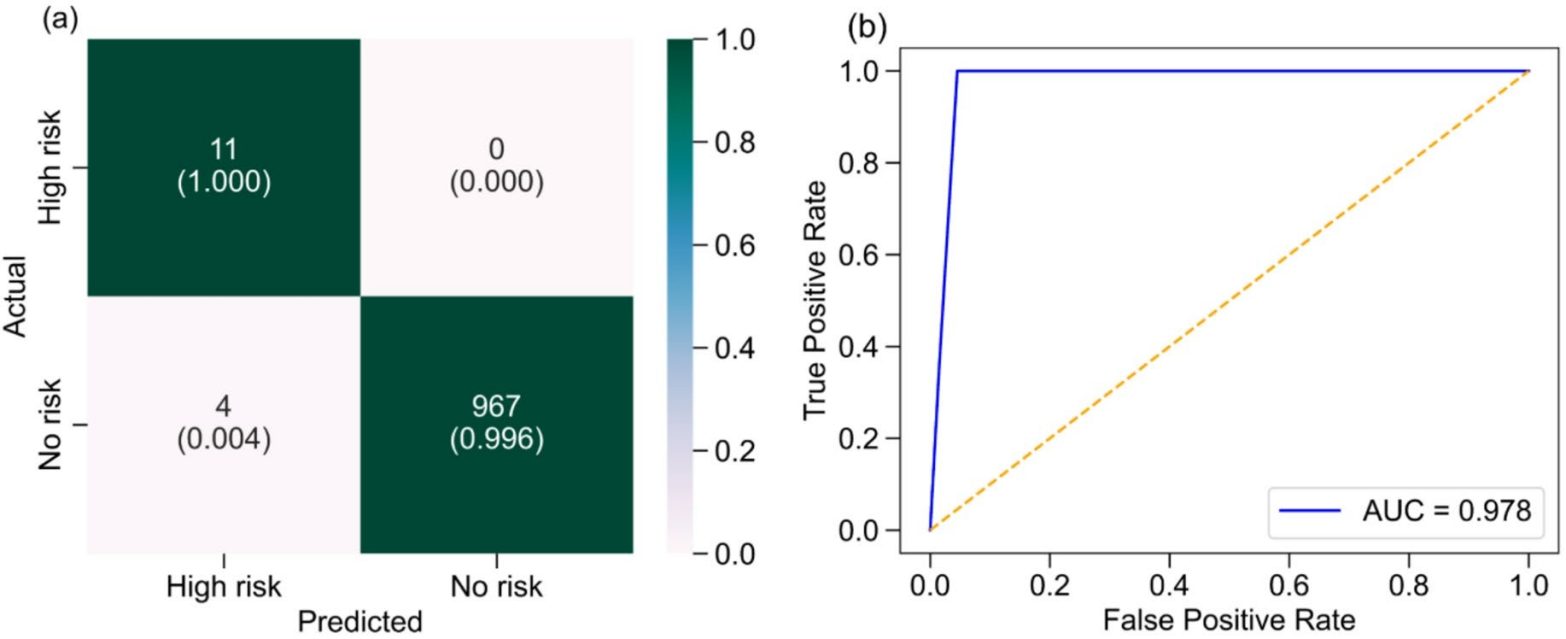
Binary classification results: (a) Recall-based confusion matrix, (b) ROC AUC.

The performance of the CJR scoring system was further evaluated using a binary classification approach. A total of 982 segmental sections were assessed for class V rock mass conditions. Sections with a high risk level correspond to actual TBM jamming locations or potential jamming zones and are therefore categorized as High Risk sections. Sections with medium, low, or no risk are categorized as No Risk sections. The binary classification results are presented in Fig. 11a.

In the binary confusion matrix (Fig. 11a), the high-risk category is considered the positive class, while the no-risk category is considered the negative class. The CJR scoring system correctly predicted all 11 actual TBM jamming sections, which indicated zero incorrectly predicted high-risk cases. Thus, the true positive (TP) rate is 1.00, and the false negative (FN) rate is 0.00. On the other hand, the actual 971 no jamming section are correctly predicted with 967 as no risk, while 4 are incorrectly predicted as high risk. This corresponds to a true negative (TN) rate of 0.996 and a false positive (FP) rate of 0.004.

In TBM risk assessment, FN predictions are more critical than FP predictions due to their direct implications for operational safety. In the case of an FN, zones with an actual high risk of TBM jamming are incorrectly classified as low- or no-risk conditions. Subsequently, the tunnelling crew may continue boring operation under normal operating parameters, which may result in unexpected TBM jamming and significant operational disruptions. Conversely, in the case of FP, ground conditions that are actually safe are classified as high-risk. Under such conditions, the tunnelling crew may adopt preventative measures, including adjustments in TBM control parameters exploiting prior experience, observational judgment, or predefined empirical adjustment ranges. In addition, detailed ground investigations and temporary stabilization measures may be carried out if judged necessary. Although FP predictions may lead to a reduced advance rate and increased operational costs due to additional investigations and operations, the tunnelling process remains within a safe operational situation.

The positive class performance was further evaluated using the receiver operating characteristic (ROC) curve and area under the curve (AUC). As shown in Fig. 11b, the x-axis represents the false positive rate (1 – specificity), indicating how often no-risk sections are misclassified as areas with high risk. The y-axis represents the true positive rate (sensitivity or recall), indicating how often actual TBM jamming sections are correctly classified as high risk. The CJR scoring system has achieved ROC AUC of 0.978, demonstrating excellent performance.

In overall, the system achieved an accuracy of 0.996, sensitivity (recall) of 1.00, specificity of 0.996, precision of 0.733, and F1-score of 0.846. The 95% CI for sensitivity and specificity were 1.00 and 0.99, respectively. These results indicate that the CJR scoring system reliably identifies high-risk sections while maintaining a low false-positive rate.

**Fig. 12:**
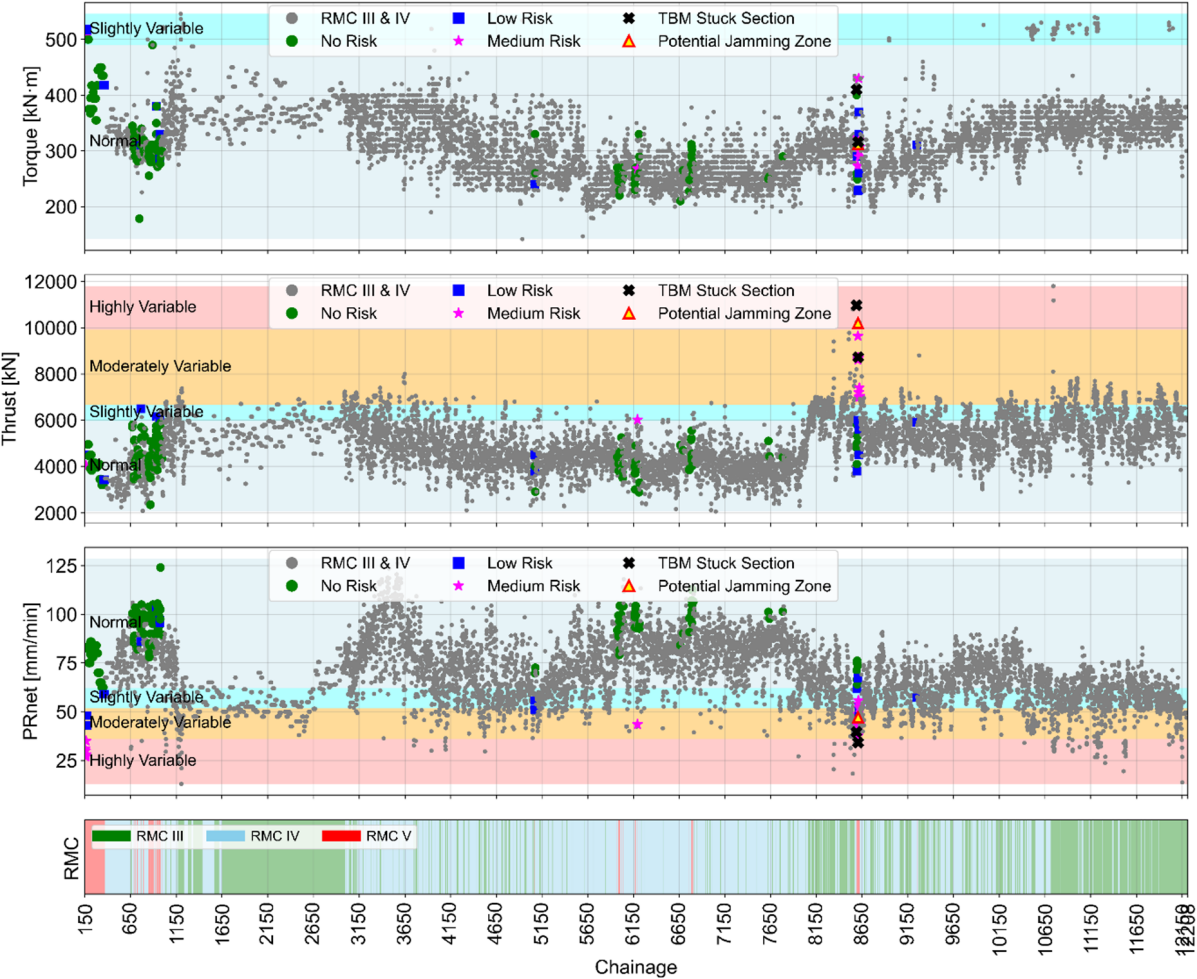
Visualization of variability scores to identify potential jamming zones in BBDM project.

**Fig. 13:**
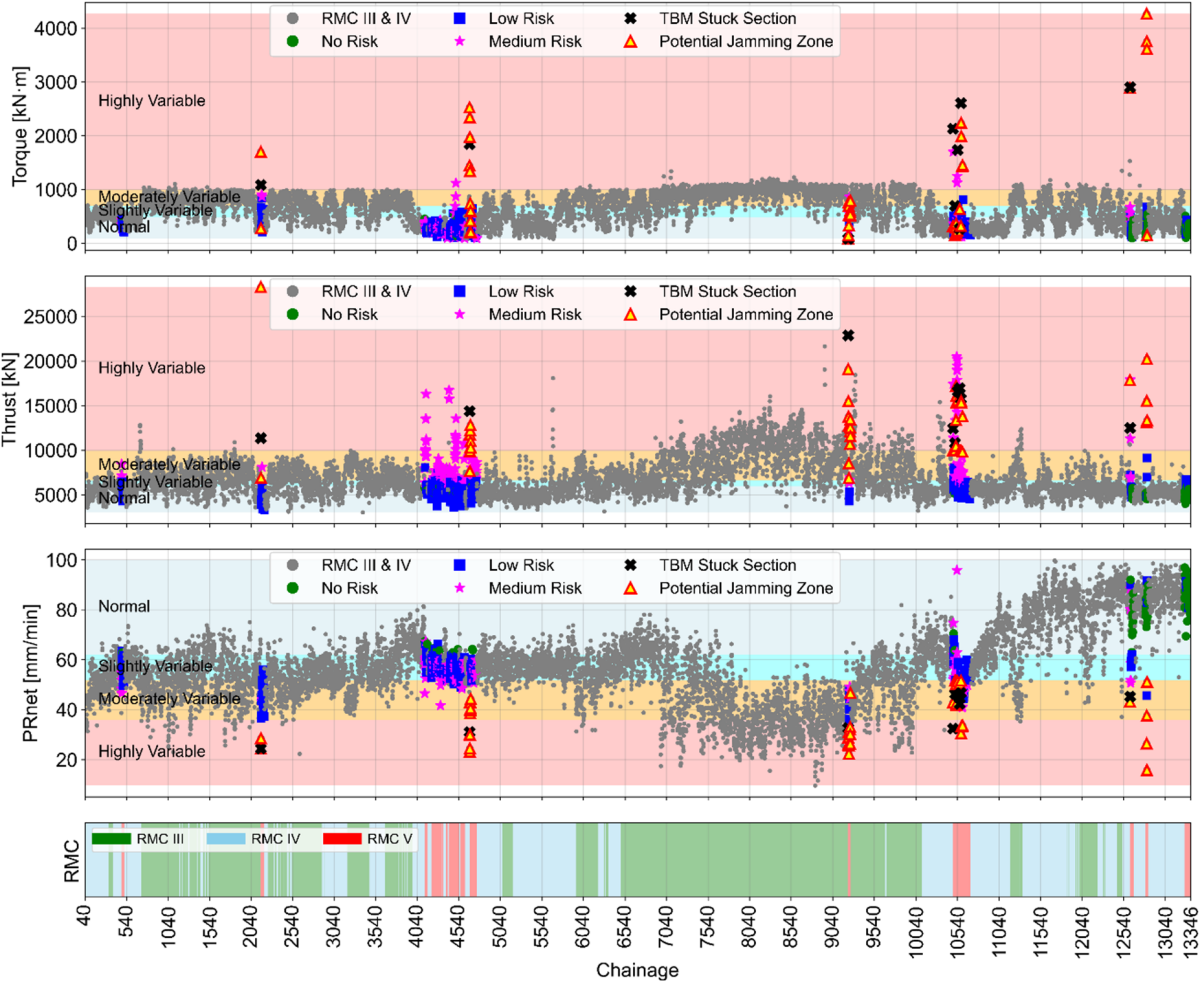
Visualization of variability scores to identify potential jamming zones in SMDM Project.

For the visualization of risk level in different tunnel sections, a color-coded scheme has been implemented along the entire tunnel alignment for both BBDM and SMDM projects. In class V, potential jamming zones are highlighted using yellow triangular markers with red borders, whereas actual TBM stuck sections are denoted by black X-shaped markers. The medium, low, and no-risk levels are represented by magenta star markers, blue square markers, and green circular markers, respectively. Additionally, data points corresponding to class IV and class V are represented by gray circles along the tunnel alignment. Furthermore, variability in torque, thrust, and PRnet is also illustrated in the background using corresponding thresholds. Percentile-based variability categories such as highly variable, moderately variable, slightly variable, and normal conditions are color-coded as red, orange, blue, and gray, respectively. A detailed risk level classification alongside corresponding data variability conditions is presented in Figs. 12 and 13.

As illustrated in Figs. 12 and 13, the torque data points largely fall within the normal variability zone, suggesting no indication of potential TBM jamming events. However, this prediction contradicts the actual tunnelling conditions and fails to identify real TBM jammed section. The variability pattern seen in thrust and PRnet provides a meaningful indication of upcoming jamming events, particularly in very poor rock mass conditions. Data points exhibiting medium to high variance correspond to high-risk conditions, which are distinctly flagged by yellow triangular markers with red borders. These alerts are typically observed at least one ring prior to the actual jamming sections. At the locations of actual TBM jamming, the high-risk conditions are marked byblack X-shaped markers.

The results demonstrate that the color-coded alarm system offers an effective early warning mechanism for the TBM tunnel crew. It supports continuous risk monitoring and enhances decision-making processes by enabling the timely implementation of preventive measures to reduce the possibility of TBM jamming.

### Empirical range of parameters

Safety is a primary prerequisite in the tunnelling process. During tunnel boring, TBM operators typically adjust control parameters based on historical performance data and real-time monitoring of TBM response to varying geological conditions. However, relying solely on prior experience and observational judgment may be insufficient in complex geological environments. As presented above, the CJR scoring system has demonstrated the ability to raise red flag warnings at least one ring in advance of potential jamming events. Notably, all recorded TBM jamming/stuck events occurred mainly in class V rock mass quality conditions.

In this regard, an empirical control system could be useful to map potentially hazardous tunnel sections. The statistical analysis indicated that medium to high risk levels are associated with moderate to high variability in geological and machine parameters under which TBM jamming was observed. Conversely, a low risk of TBM jamming was found in cases of slight variability. Therefore, operational conditions ranging from normal to slightly variable can be considered safe for tunnelling operations, and a threshold up to slight variability may be used to define safe working conditions. Based on the findings of this research, an empirical control range for key TBM input and response parameters has been established between the 25th and 75th percentile values derived from the cross-project TBM database, which are summarized in Table 12.

**Table 12:** Empirical range for TBM control and response parameters with corresponding rock mass class.

Rock mass quality class	Tunnelling parameter				
	Control Parameter			Response Parameter	
	CRS (rpm)	PRchd (mm/rev)	PRnet (mm/min)	Torque (kN.m)	Thrust (kN)
Fair rock mass (class III)	7.0–7.25.0.25	6.40–8.70	46.10–61.40	352.50–880	5501–7598
Poor rock mass (class IV)	7.0–8.0	8.20–11.10	57.50–80.40	270–460	4367.25–5815
Very poor rock mass (class V)	5.0–7.0	9.38–13.10	54.70–83.10	241–394	4264.2–5886.75.2.75

Utilizing these empirically defined ranges can support TBM operators in making adaptive adjustments and data-informed decisions during tunnel excavation in the challenging geological conditions of the Himalaya.

## Conclusions

This study proposed a novel ML-based framework for predicting TBM performance in the complex geological settings of the Himalayan region utilizing cross-project database. These datasets encompassed diverse geological conditions and varying TBM configurations and were analyzed using ensemble models such as RF, bagging, XGBoost, stacking ensemble, and ANN regression models. The ML models exhibited high predictive accuracy within-project datasets, achieving coefficients of determination (R²) between 0.957 and 0.980 for training sets, and between 0.936 and 0.938 for testing sets. However, model performance declined when it was tested on cross-project data, particularly when models trained on the BBDM dataset were applied to the Siwalik section of the SMDM project. Despite similar geological settings, differences in TBM configurations led to R² values ranging from 0.416 to 0.838. Among the tested models, stacking, bagging, and XGBoost demonstrated fairly good cross-project performance, with R² values of 0.749, 0.823, and 0.838, respectively; however, the overall generalization remained insufficient.

To enhance model efficiency, datasets from the Siwalik regions of both projects were merged to create a combined training database. The models were then tested on the Lesser Himalayan section of SMDM project. This strategy significantly improved the model robustness, with R² values ranging from 0.895 to 0.972. Encouraged by this improvement, the models were trained and tested on a combined, stratified dataset using 5-fold cross-validation. The models exhibit average R² values on test sets ranging from 0.960 to 0.965, along with low loss function values and good CI across all folds. These results confirm that this approach significantly improves the robustness of ML models for predicting PRnet across different geological conditions and machine configurations.

Furthermore, the feature importance analysis using SHAP values revealed that the TBM PRnet is positively influenced by control parameters such as PRchd and CRS. In contrast, response parameters like torque and thrust exhibited a negative influence on PRnet. Geological parameters, particularly rock mass quality, also played a significant role. Based on these insights, a CJR scoring system was developed using a percentile-based statistical approach, incorporating PRnet, TBM response parameters, and rock mass quality conditions. The system effectively identified both actual TBM jamming areas and potential jamming areas, providing early warning signals for at least one ring (~ 1.5 m) in advance of potential TBM stuck events. Empirical ranges for TBM control and response parameters were also established for different geological conditions to aid in real-time operational adjustments during TBM tunnelling.

In summary, the proposed ML framework demonstrated robust predictive performance for TBM advancement and jamming risk across projects with diverse geology and machine configurations. The integration of performance prediction with proactive jamming risk assessment offers a valuable tool for real-time TBM operation optimization while tunnelling through challenging geotectonic conditions like in the Himalayan region of Nepal and in similar geological conditions around the world.

## Limitations and future work

This study demonstrates the potential of supervised ML techniques for predicting TBM PRnet and assessing jamming risk using a cross-project double-shield TBM database from Siwalik and Lesser Himalayan geological formations. The proposed models show strong predictive performance. However, database from two TBM projects passing through Siwalik and Lesser Himalayan rock mass conditionswere employed. Hence, the proposed models are relevant to similar geological conditions.

The authors recommend to expand research using more datasets from other TBM projects. In addition, it is fruitful to include data from different TBM types and varying geological formations. Further, the computational requirements, temporal efficiency, and real-time implementation challenges of the model need to be evaluated by exploring a wider range of ML techniques.

## Data Availability

The datasets generated and/or analysed during the current study are not publicly available due to project confidentiality but are available from the corresponding author on reasonable request.
